# Comparison of the Relationship between Cerebral White Matter and Grey Matter in Normal Dogs and Dogs with Lateral Ventricular Enlargement

**DOI:** 10.1371/journal.pone.0124174

**Published:** 2015-05-04

**Authors:** Martin J. Schmidt, Steffi Laubner, Malgorzata Kolecka, Klaus Failing, Andreas Moritz, Martin Kramer, Nele Ondreka

**Affiliations:** 1 Department of Veterinary Clinical Sciences, Clinic for Small Animals, Justus-Liebig-University-Giessen, Giessen, Germany; 2 Unit for Biomathematics and Data Processing, Faculty of Veterinary Medicine, Justus Liebig-University-Giessen, Giessen, Germany; University of Surrey, UNITED KINGDOM

## Abstract

Large cerebral ventricles are a frequent finding in brains of dogs with brachycephalic skull conformation, in comparison with mesaticephalic dogs. It remains unclear whether oversized ventricles represent a normal variant or a pathological condition in brachycephalic dogs. There is a distinct relationship between white matter and grey matter in the cerebrum of all eutherian mammals. The aim of this study was to determine if this physiological proportion between white matter and grey matter of the forebrain still exists in brachycephalic dogs with oversized ventricles. The relative cerebral grey matter, white matter and cerebrospinal fluid volume in dogs were determined based on magnetic-resonance-imaging datasets using graphical software. In an analysis of covariance (ANCOVA) using body mass as the covariate, the adjusted means of the brain tissue volumes of two groups of dogs were compared. Group 1 included 37 mesaticephalic dogs of different sizes with no apparent changes in brain morphology, and subjectively normal ventricle size. Group 2 included 35 brachycephalic dogs in which subjectively enlarged cerebral ventricles were noted as an incidental finding in their magnetic-resonance-imaging examination. Whereas no significant different adjusted means of the grey matter could be determined, the group of brachycephalic dogs had significantly larger adjusted means of lateral cerebral ventricles and significantly less adjusted means of relative white matter volume. This indicates that brachycephalic dogs with subjective ventriculomegaly have less white matter, as expected based on their body weight and cerebral volume. Our study suggests that ventriculomegaly in brachycephalic dogs is not a normal variant of ventricular volume. Based on the changes in the relative proportion of WM and CSF volume, and the unchanged GM proportions in dogs with ventriculomegaly, we rather suggest that distension of the lateral ventricles might be the underlying cause of pressure related periventricular loss of white matter tissue, as occurs in internal hydrocephalus.

## Introduction

Despite the high diversity of head conformation in dog breeds, differences in canine brain morphology are comparably small [1–3]. Due to the restricted longitudinal skull growth in brachycephalic dogs their brains show reduced longitudinal extension [2, 4]. As a result the external morphology resembles the juvenile state of canine brains, showing a wide and stocky appearance and ventrally orientated olfactory bulbs [2, 5]. Nevertheless, the general morphology of the brain including the cortical pattern of sulci and gyri is the same amongst dogs with different head conformations [1, 3]. However, a frequent finding in brachycephalic dog brains is relatively large lateral cerebral ventricles in comparison with mesaticephalic dogs [6–8]. It has been widely accepted that this increase in ventricular volume is not associated with clinical signs and that most small-breed dogs normally have large lateral ventricles as a breed characteristic and are not truly hydrocephalic [9–12]. As most brachycephalic dogs are small toy-breeds it has been suggested that ventricular size follows negative allometric growth principles [13]. The term “constitutional hydrocephalus” has been used to describe the common association of large ventricles with short stature in brachycephalic dogs [14]. On the other hand the terms “ventricular enlargement” and “ventriculomegaly” have been used to describe the large ventricles in these breeds, which would imply a pathological condition to some extent [11, 15, 16].

Several studies aiming to determine the physiological ventricular dimensions in dogs suggested that ventricular size can vary in individual dogs of the same breed and size [17, 18]. However, the following morphological rule conflict with this suggestion: The expansion of the cerebral cortex is one of the most distinctive morphological features of the mammalian brain. Its functional organization is subject to defined spatial, electro-physiological and metabolic constraints within a limited volume [19]. In the process of evolution this has led to brains with a defined relationship between white matter (WM) and grey matter (GM) mass [19].

As brain size has increased during evolution, the various parts of the brain have not simply increased proportionally. In brains of large animals the WM mass in the neocortex has increased disproportionately relative to GM [20–22]. This reflects changes in the diameter of axons, with larger brains having thicker axons and thicker myelin sheaths [23] and more cortico-cortical connections per neuron [20]. Both, the defined relationship between WM and GM and its defined increase with body size are universal in all mammalian species (eutheria) and can be expressed by allometric scaling laws [19, 20, 24, 25]. We would therefore expect a characteristic relationship between WM and GM mass to exist in different dog breeds and that WM would also hyperscale with increasing body weight in dogs. If large ventricles would represent a normal morphologic variation, a WM/GM relationship similar to dogs with normal ventricles should be preserved in dog brains with large ventricular volume. Aberrations from the WM/GM relationship would indicate that ventricular enlargement is associated with a reduction of brain tissue as the sum of brain tissues and CSF is constant (Monroe-Kellie-doctrine). This hypothesis states that CSF and brain tissue (including blood vessels) create a state of volume equilibrium. Any increase in volume of one of the cranial constituents must be compensated by a decrease in volume of another. In clinical analyses of brain images of dogs with subjectively normal lateral ventricles and dogs with ventriculomegaly it was our observation that the periventricular WM is reduced in individuals with larger ventricles, as occurs in hydrocephalus. We therefore hypothesize that the relationship between WM and GM is decreased in dogs with “oversized” ventricles.

### Ethics statement

The MRI data were obtained for diagnostic purposes and retrospectively analyzed in this study. Therefore approval from the ethics committee of the Justus Liebig University and government of the county Hessen (Regierungspräsidium) was not sought as it is the policy of the ethics committee not to subject retrospective studies of images stored in the archive to ethical review. The “Deutsches Tierschutzgesetz” (German Animal Protection Law) does not request a written waiver for retrospective studies. MR-imaging data of five healthy Beagles were also taken from an experimental study of brain diffusion and perfusion in normal dogs. This study was carried out in strict accordance with the recommendations in the Guidelines for Care and Use of Laboratory Animals of the German Animal Protection Law. The protocol was approved by the Committee on the Ethics of Animal Experiments of the Justus Liebig University Giessen and Regierungspräsidium Hessen (Permit Number: V54-19c2015(I)Gi18/17 No. 78/2011).

## Materials and Methods

### Animals

**Fig 1:**
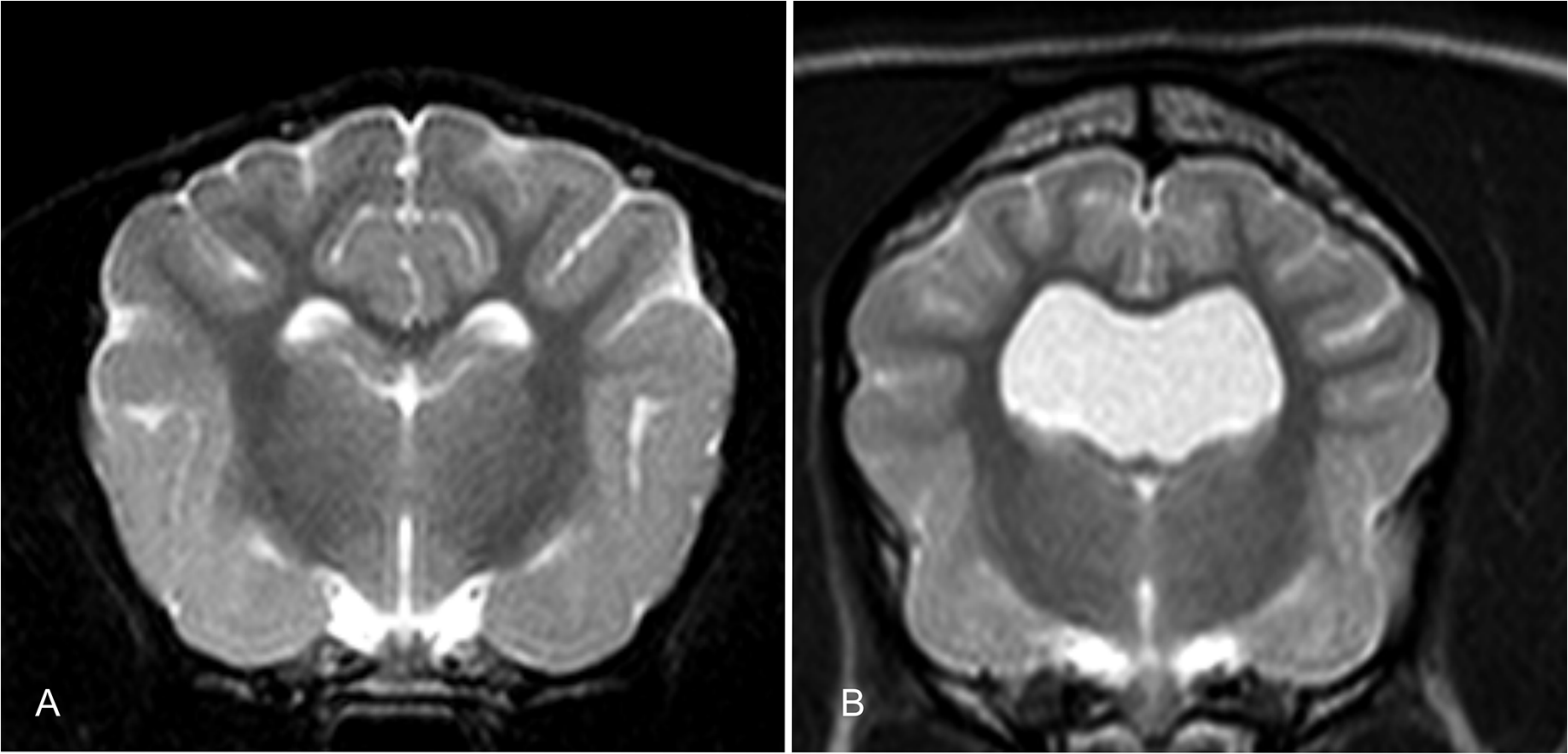
Comparison of a canine brain with normal lateral cerebral ventricles (A) and enlarged ventricles (B).

The archive of MRI scans of the Justus Liebig University (JLU), Giessen, Germany, was searched retrospectively for MR-imaging reports including the diagnoses “primary or idiopathic epilepsy”, “within normal limits” and “ventriculomegaly” or “enlarged ventricles”. The presence of ventriculomegaly was based on the following criteria: The majority of dogs have very narrow and slit-like horns of the lateral ventricles. In the finding of large ventricles/ventriculomegaly, the interpreter subjectively noted a greater proportion of the intracranial volume occupied by the lateral ventricles. The closely spaced walls of the temporal horns and/or the olfactory recesses were separated by CSF in these brains and the lacking septum pellucidum created a large connection between the first and second ventricle (Fig 1) [26]. MRI reports for each series were obtained by board certified radiologists at the JLU. These dog brain series were assessed for suitability of inclusion in this study using the following criteria. None of the patients was allowed to show evidence of space occupying lesions or other morphological alterations of the brain parenchyma. Transverse scans had to include the whole brain from the cribriform plate rostrally to the first cervical spinal cord segment caudally. Series with inadequate image contrast and spatial resolution or incomplete transverse series were excluded from the study. The finding of “ventriculomegaly” or “enlarged ventricles” had to be judged as an incidental finding. “The breed, gender, age, and body weight of the dog at the time of scanning were recorded, and dogs that were between 1 and 6 years of age and up to 17 kg in bodyweight were included.” Subjects were divided into the following groups: Group one included dogs whose brain and lateral ventricles were assessed as normal by the diagnosis of “within normal limits”. Group two included dogs in which a dilatation of the lateral ventricles was noted on interpretation of the images and ventriculomegaly or enlarged ventricles were recorded as an incidental finding.

### Imaging technique

Imaging was performed using a 1 Tesla MRI scanner (Gyroscan Intera, Phillips, Hamburg, Germany). From the whole MR dataset T2-weighted transverse images of the head were chosen for image segmentation. Images were obtained using T2-Turbospin echo sequences (TE: 120 ms, TR: 2900 ms). Slice thickness varied from 2–3 mm. The field of view measured 180 x 180 mm in small dogs and 210 x 210 mm in large dogs. The matrix was 288 x 288 in small dogs and 384 x 384 in large dogs leading to an in-plane pixel size between 0.625 x 0.625 mm and 0.54 x 0.54 mm.

### Morphometric procedures

**Fig 2:**
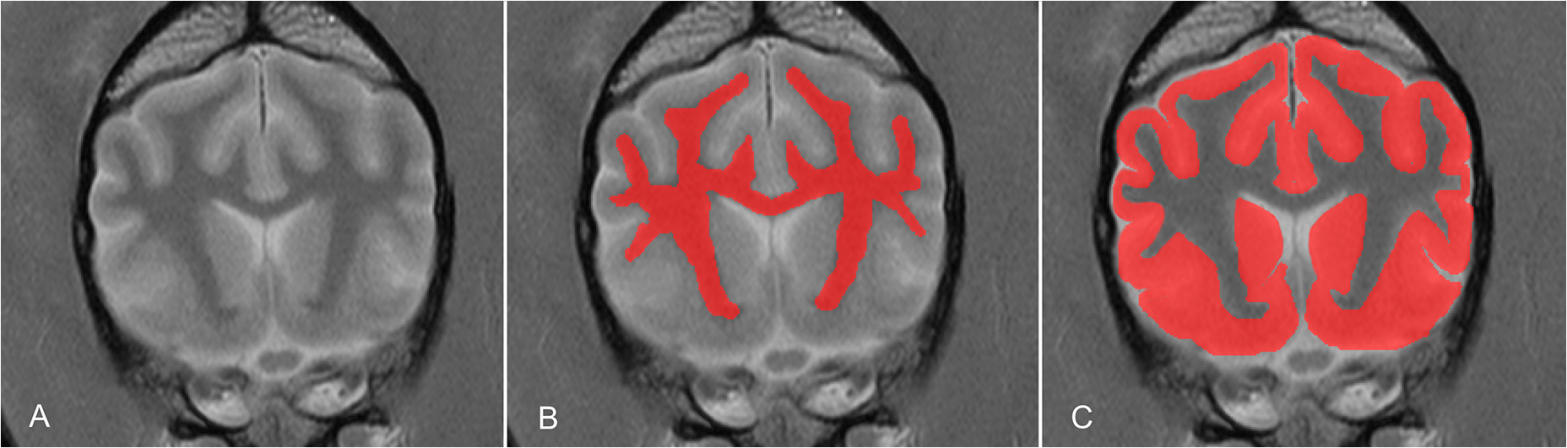
Volume determination based on MRI-datasets. Image segmentation of white matter and grey matter using manual segmentation on a slice-by-slice basis from transverse images. Each tissue of interest is labelled red and thereby assigned to a group (mask). All masks are then assembled and the tissues can be depicted in volume form.

The mass of GM and WM in the brain was determined based on MRI datasets as described previously [15, 27–30]. Study numbers were assigned to dogs so that the observer was blinded to the signalment of the dog and its previous image interpretation. All segmentation procedures were performed by one investigator. Image processing for volume rendering was achieved using specialized graphical software (AMIRA, Mercury Computers Systems, Berlin, Germany), which allows manual image segmentation on a slice-by-slice basis. This program allows interactive segmentation in all image planes (“four-viewer mode”). In this mode, three 2D viewers with different reconstructed orientations and an additional 3D viewer are displayed in which segmentation can be simultaneously performed. Image segmentation in this context describes the manual tracing of the WM and GM in each image based on their differential signal intensity. All voxels corresponding to a single anatomical structure in the images are selected and assigned to the same value in the mask. The final mask thus contains information about all selected anatomical structures and in combination with the original data and polygonal surface reconstruction algorithms, allows the visualization of different structures in the images [29]. Segmentation was performed manually in transverse orientation from individual slices (Fig 2). Only the GM and WM of the cerebrum (neocortex and paleocortex) including the basal ganglia were determined. The ammon`s horn (archicortex) was excluded because nine dogs were examined due to seizures, which can lead to volume loss of this structure.

**Fig 3:**
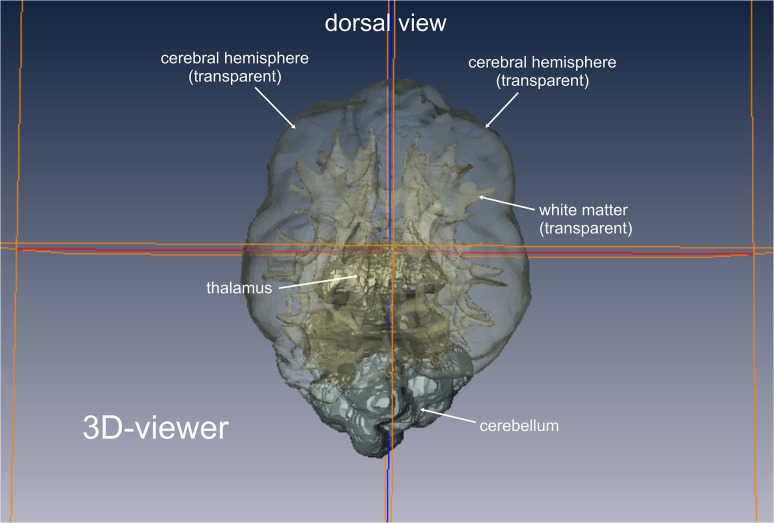
Volume rendering of brain tissues of interest. 3D viewer mode of the graphical software AMIRA. The voxels of the tissue of interest (white matter/grey matter) of each slice have been assembled and are now displayed as a 3D model. Each tissue can be displayed solid or transparent. The localizer lines support the segmentation process. As they are displayed in both the 2D images and the 3D model, the thalamus, medulla and cerebellum can be accurately separated from the volume of interest.

**Fig 4:**
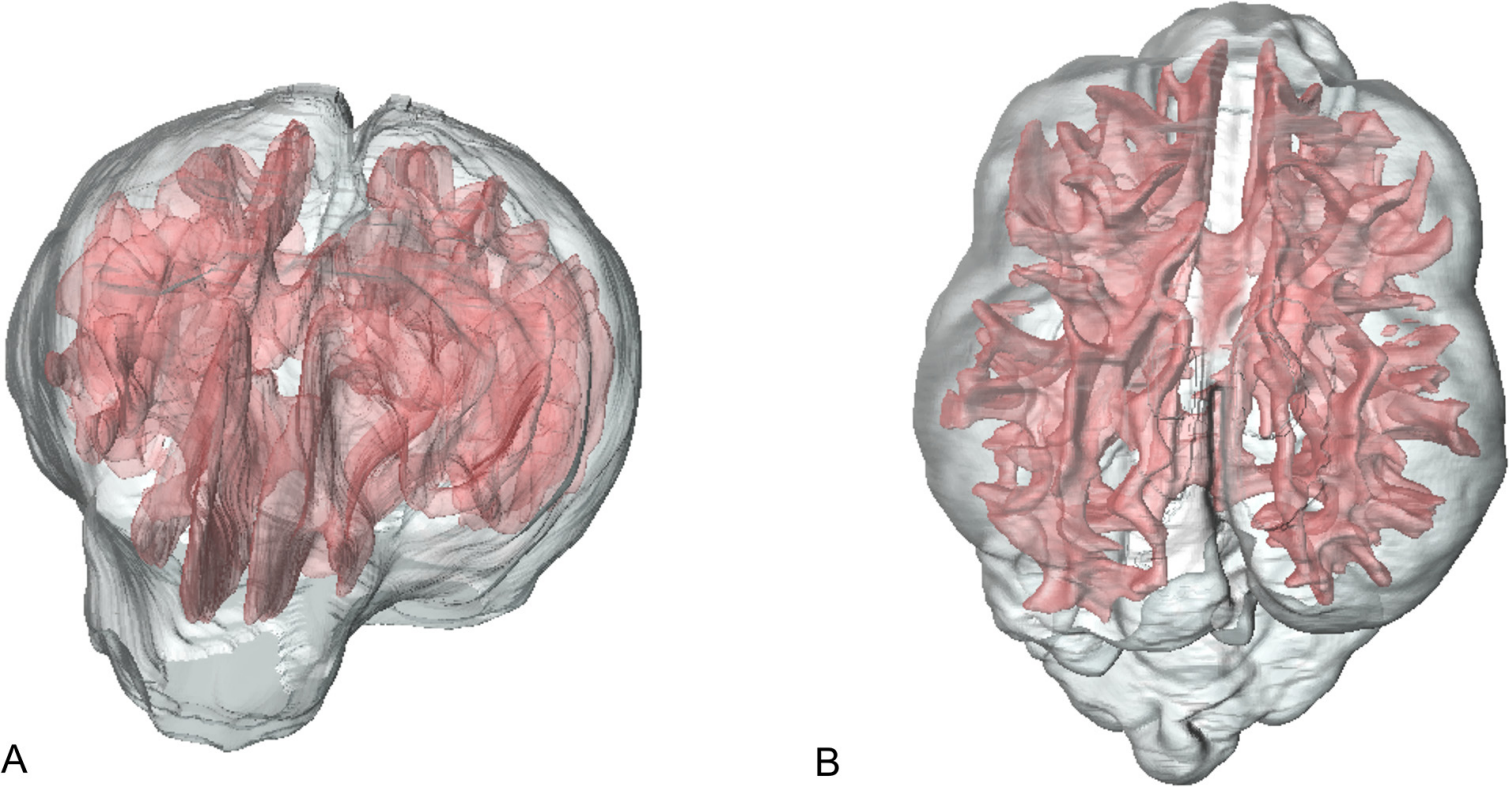
Volume models of grey matter and white matter. Frontal (A) and dorsal view of a 3D model of the brain of a Jack Russell Terrier. The white matter is labelled in red and the grey matter is transparent grey.

In the first step the cerebellum, brainstem, thalamus and hippocampus were segmented, assigned to a mask and excluded from the volume of interest (Fig 3). As the WM of the cerebral hemispheres, i.e. the internal capsules, merges into the thalamus running caudad towards the brainstem, a perpendicular line connecting the caudal border of the optic chiasm and the rostral border of the intermediate mass was drawn in reconstructed sagittal images. This line was automatically represented in all other imaging planes including the 3D model and was used to delineate the internal capsule transition from the cerebral hemispheres to the diencephalon. WM caudal to this line was not included. The lateral ventricles were segmented in a third step. The lateral ventricles included the Monroe`s foramen. The third and fourth ventricle was not included. The segmented WM GM and CSF partitions were calculated and graphically presented by the program (Fig 4 and S1 Table). The calculated volumes were put into relation to the whole cerebral volume and expressed as the relative CSF volume and relative GM- and WM volume. Furthermore the relationship between WM and GM was expressed as the WM/GM ratio.

### Statistical analysis

Statistical analysis was performed using the commercial statistical software package BMDP (BMDP Statistical Software, Inc., Los Angeles, USA). To test homogeneity of the groups the relative frequency of male and female animals in the groups was compared using Fisher`s exact test.

Relating the group comparison of target variables, in a first step the relative GM volume, the relative WM volume, relative CSF-volume and the WM/GM ratio was plotted against the body weight of the animals. Then the residuals of the linear regression model including group effects were checked for normality using a Q-Q-plot. If the conditions for its application are given the one-way analysis of covariance (ANCOVA) was used to assess the significance of the difference between the groups including the relationship between the independent variable body weight (X-axis) and the dependent variables relative GM-, WM- and CSF-volume as well as WM/GM ratio, (Y-axis). The slopes of the regression lines were compared between groups, testing the null hypothesis that for a special dependent variable the slopes are all identical (the lines are parallel). In a second part of the analysis the adjusted means (sample means adjusted for the common mean body weight and a common slope) of all groups were calculated and checked for significant differences between groups [30].

## Results

### Animals

The dogs included in this study had been examined for various reasons presented in Table 1 together with breed and bodyweight. Five normal beagles of different body weight that were scanned as a part of another study were included this investigation. Group one included 37 mesaticephalic dogs. Their bodyweight ranged from 2.1 to 17 kg. Their median age was 3.0 years. Twenty of the dogs were male, 17 were female.

Group two included 35 brachycephalic dogs. Their bodyweight ranged from 1.8 to 17 kg. Their median age was 3.6 years. Sixteen dogs were male, 19 were female. A Fisher exact test revealed no differences in the frequency distribution of gender between groups (p = 0.145).

**Table 1:** Breed, body weight and diagnosis of the dogs included in groups one and two.

Group1:	Breed	Bodyweight	Indication for MRI/ Final diagnosis
**1**	Poodle	3 kg	Optic neuritis
**2**	Wirehaired dachshund	4.5 kg	Behavioral changes / aggression
**3**	Dachshund	2.1 kg	Idiopathic epilepsy
**4**	Beagle	10 kg	Study of brain perfusion
**5**	Beagle	11 kg	Study of brain perfusion
**6**	Beagle	10 kg	Study of brain perfusion
**7**	Beagle	9 kg	Study of brain perfusion
**8**	Beagle	9.4 kg	Study of brain perfusion
**9**	Mixed breed	3 kg	Otitis media
**10**	Dachshund	2.2 kg	Idiopathic epilepsy
**11**	Dachshund	2.8 kg	Idiopathic epilepsy
**12**	Miniature schnauzer	6 kg	Behavioral abnormality /aggression
**13**	West Highland White terrier	9 kg	Otitis externa
**14**	Wirehaired dachshund	7 kg	Otitis media
**15**	Mixed breed	3 kg	Otitis media
**16**	Mixed breed	3.2 kg	Retrobulbar abscess
**17**	Mixed breed	9.4 kg	Intraorbital inflammation
**18**	Beagle	9.4 kg	Retrobulbar tumor
**19**	Jack Russel terrier	5 kg	Nasal tumor
**20**	Norfolk terrier	6.5 kg	Dorsal dens angulation
**21**	Miniature pinscher	4.8 kg	Nasal tumor
**22**	Jack Russel terrier	9 kg	Masticatory myositis
**23**	Cocker spaniel	13 kg	Trigeminal nerve neuritis
**24**	Wirehaired dachshund	8 kg	Behavioral abnormality /aggression
**25**	Nova Scotia duck tolling retriever	17 kg	Pain of undetermined origin
**26**	Schnauzer	14.5	Otitis media
**27**	Austrian hound	17 kg	Rhinitis
**28**	Cocker Spaniel	12.5 kg	Otitis media
**29**	Schnauzer	14 kg	Nasopharyngeal mass
**30**	Beagle	14.5	Facial nerve paralysis
**31**	Mixed breed	10 kg	Otitis media
**32**	Mixed breed	7 kg	Idiopathic vestibular syndrome
**33**	Dachshund	2.4 kg	Retropharyngeal abscess
**34**	Miniature Pinscher	6 kg	Idiopathic epilepsy
**35**	Mixed breed	15 kg	Trigeminal nerve neuritis
**36**	Poodle	2.9 kg	Idiopathic epilepsy
**37**	Beagle	8.5 kg	Retrobulbar abscess
**Group 2**	Breed	Bodyweight	Indication for MRI/ Final diagnosis
**1**	Chihuahua	1 kg	Neck pain, atlanto-axial subluxation
**2**	Chihuahua	1 kg	Neck pain, atlanto-axial subluxation
**3**	Chihuahua	2 kg	Neck pain, dorsal dens angulation
**4**	Bolonka Zwetna	2 kg	Atlantoaxial instability
**5**	Papillion	3.2 kg	Atlantoaxial instability
**6**	Shih Tzu	5.2 kg	Otitis media
**7**	Shih Tzu	5.5 kg	Pain of undetermined origin
**8**	Pug dog	8.5 kg	Masticatory myositis
**9**	Pug dog	13 kg	Otitis media
**10**	Pug dog	8.4 kg	Compulsive obsessive behavior
**11**	Pug dog	8.7 kg	Dorsal dens angulation
**12**	French Bulldog	8.5 kg	Otitis media
**13**	French Bulldog	13 kg	Otitis media
**14**	French Bulldog	13.5 kg	Optic neuritis
**15**	French Bulldog	9 kg	Deafness
**16**	French Bulldog	8,9 kg	Neck pain—arachnoid cyst C2
**17**	French Bulldog	11 kg	Otitis media/interna
**18**	French Bulldog	10 kg	Retropharyngeal mass
**19**	French Bulldog	10 kg	Otitis media
**20**	French Bulldog	12.5 kg	Otitis media
**21**	Shih Tzu	6.7 kg	Retrobulbar abscess
**22**	Shih Tzu	7.6 kg	Optic neuritis
**23**	Tibet terrier	5 kg	Seizures—cardiac syncopes
**24**	Yorkshire terrier	4.3 kg	Pain of undetermined origin
**25**	CKCS	11 kg	Retropharyngeal abscess
**26**	CKCS	7.5 kg	Lymphoma trigeminal nerve (extrancranial)
**27**	CKCS	5 kg	Breeding selection syringomyelia
**28**	CKCS	8 kg	Breeding selection syringomyelia
**29**	CKCS	12 kg	Breeding selection syringomyelia
**30**	CKCS	10 kg	Breeding selection syringomyelia
**31**	CKCS	14.5 kg	Otitis media
**32**	Yorkshire terrier	3.5 kg	Pain of undetermined origin
**33**	Pekingese	6.1 kg	Otitis media
**34**	Pekingese	7.6 kg	Otitis media
**35**	English bulldog	17 kg	Head bobbing

**Fig 5:**
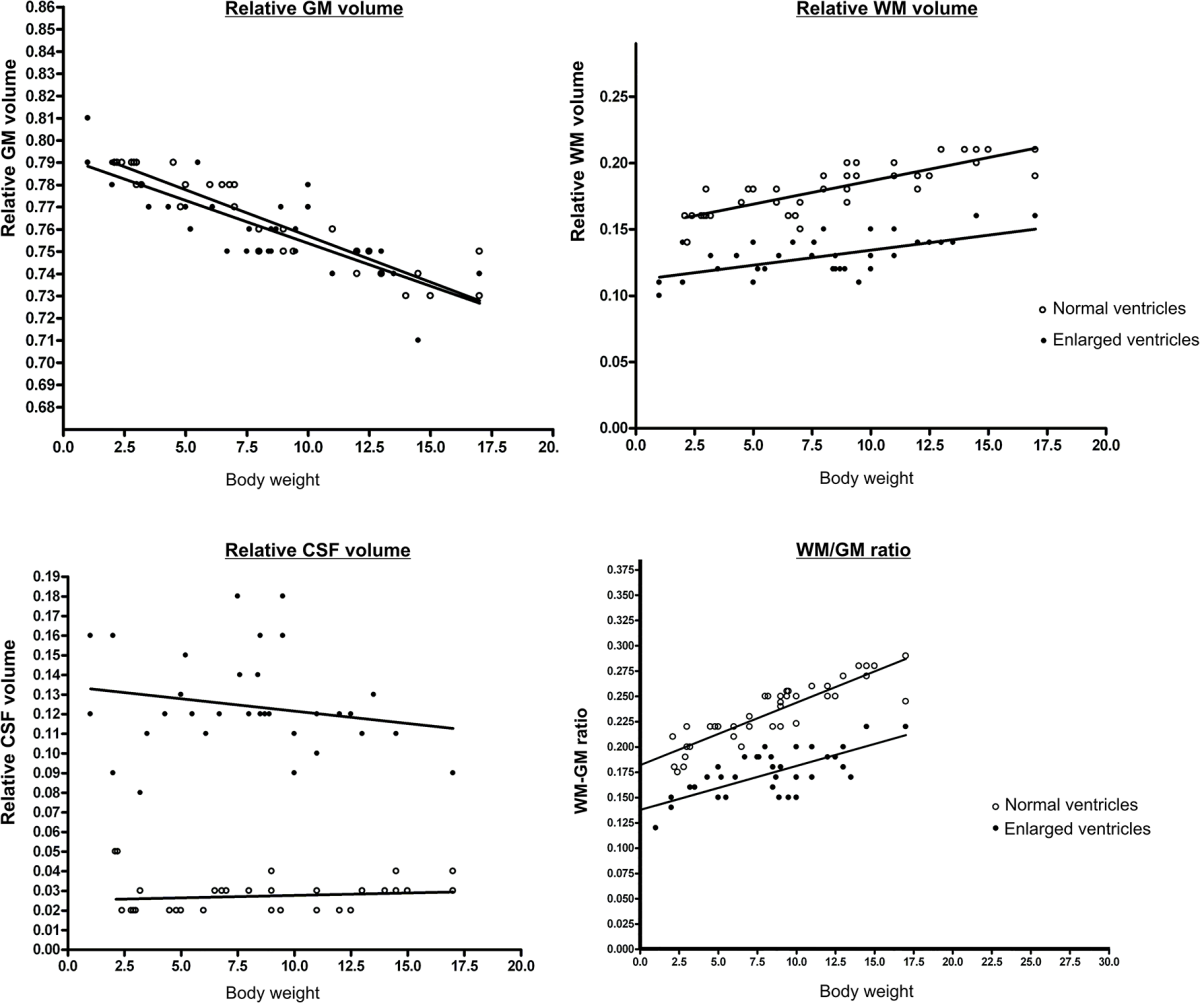
Linear regression model analysis of the changes in relative grey matter (GM), white matter (WM)- and CSF volume and the WM/GMratio with increasing bodyweight in dogs. The relative grey matter (GM), white matter (WM), and CSF volume of the lateral venmtricles as well as the WM/GM-ratio is plotted against the bodyweight in two groups of dogs. The open circles represent dogs with subjectively normal ventricles, the filled circles represent dogs with enlarged ventricles. The adjusted means in the middle of the regression lines of the groups differs significantly.

The results of the analysis with the ANCOVA model to are presented in Table 2 and Fig 5. All parameters show a significant correlation to bodyweight. Dogs with ventriculomegaly have a significantly higher adjusted means of the relative CSF volume. The WM/GMratio is significantly decreased in dogs with ventriculomegaly. Whereas the adjusted means of the relative GM was not significantly different between groups the adjusted means of the relative WM volume was significantly decreased in the groups with ventriculomegaly. Therefore, the model predicts that at the same bodyweight dogs with oversized ventricles have less WM than dogs with subjectively normal lateral ventricles.

**Table 2:** Results of the one-way analysis of covariance.

variable	group	Adjusted means (atBW = 8.26)± SEM	Equality of the adjusted means	Common regression coefficient	p-value	equalitiy of slopes: estimates	equalitiy of slopes: p-values	r /R2
**Relative GM volume**	1	0.7645(±0.0016)	p = 0.061	-0.0041	<0.0001	-0.0043	0.344	0.919/0.844
**Relative GM volume**	2	0.7601(±0.0017)				-0.0038		0.809/0.654
**Relative WM volume**	1	0.1767(±0.0018)	p<0.0001	0.0033	< 0.0001	0.0037	0.172	0.843/0.71
**Relative WM volume**	2	0.1326(±0.0019)				0.0028		0.698/0.487
**Relative CSF volume**	1	0.0597(±0.0017)	p<0.0001	0.0010	0.002	0.0008	0.489	0.441/0.194
**Relative CSF volume**	2	0.1071(±0.0018)				0.0012		0.352/0.123
**WM/GM ratio**	1	0.2320(±0.0027)	p<0.0001	0.0055	< 0.0001	0.0063	0.052	0.875/0.765
**WM/GM ratio**	2	0.1736(±0.0028)				0.0044		0.729/0.531

The table presents the results of the analysis of covariance testing the equality of slopes from the regression to the body weight (BW), the global relevance of the BW as a covariate (common slope different from zero) and the equality of adjusted means at mean BW = 8.26 kg between the groups. The adjusted means are presented including the standard error of means (SEM)

## Discussion

The dimensions of the cerebral ventricular volume in dogs have been reported to vary [17, 18]. In brachycephalic dogs in particular, the ventricles can be larger than expected. This has been referred to as ventriculomegaly to differentiate this condition from clinically relevant internal hydrocephalus. However, the designation of normal ventricles and enlarged ventricles remain poorly defined and are usually assessed subjectively [26].

It is unclear whether ventriculomegaly should be interpreted as one variant of a spectrum of ventricular dimensions in dogs or rather as a pathological condition. It was our hypothesis that the larger ventricles in brachycephalic dogs are a pathological condition as indicated by concurrent presence of WM loss due to ventricular distention as found in internal hydrocephalus. To investigate this hypothesis we examined the relative WM, GM, and CSF volume in dogs with different ventricular dimensions as well as their WM/GM ratio in proportion to their body weight and cerebral volume. The age of the animal has an important influence on the volumes of neural tissues, myelination and ventricular size. As cortical atrophy and ventricular enlargement have been identified as characteristic features of the aged canine brain [31], only dogs < 6 years of age were included in the study. Brain volume, myelination and ventricular volume change during brain growth [6, 32–34]. Therefore, only dogs with a minimum age of 12 months were included. Testosterone levels are positively correlated with myelinogenesis [35]. This could lead to larger white matter volumes in male dogs. The potential influences of sexual dimorphism can be ignored in our study as the proportion of male and female dogs did not differ significantly between groups.

Epilepsy was one reason for MRI examination in 9 dogs of our control group. Although seizure activity can lead to brain damage, epileptic dogs have previously been used to study brain volumetrics [26, 28] as damage only occurs at the microscopic level [36, 37]. However, the hippocampus was a priori excluded from grey matter volume because variable loss of hippocampal volume identified on MR-images has been linked to seizure activity in dogs [38, 39].

MRI-based volume measurements of the brain parts are routinely performed in human and veterinary medicine [13, 15, 28, 30, 38,40, 41]. The accuracy of the technique even for small volumes has been proven in both veterinary [8, 17, 42] and human neuroradiology [42, 43]. However, manual segmentation approaches can be subject to investigator bias. The validity of our approach would have further increased with multiple examinations but we refrained from the determination of interobserver-variability as the manual segmentation process is extremely time consuming. This is the main limitation of the present study. Large and small dog heads were scanned using different receiver coils (knee coil, surface coil). It is possible that using different coils could have caused a bias in our measurements due to random differences in contrast or resolution. We only included image sets with sufficient resolution and contrast.

Our approach to comparing WM and GM volume in dogs followed the assumption of a basic uniformity of the neocortex and its connections in all dog breeds. We did not consider other non-allometric factors based on selection related to behavioural and environmental factors affecting brain size independently from selection for body size by breeders. Some breeds have been selectively bred for the “quality of learning,” but this has been upstaged in other breeds in favour of other characteristics. Mesaticephalic dogs have been reported to have higher trainability than brachycephalic dogs, which may have an influence on the number and connectivity of neurons [44]. This could influence the calculated relations if such dogs have more GM or WM at a given bodyweight (grade shift of brain mass), especially given that group two exclusively included brachycephalic dogs. Analysis of large homogenous groups of dogs would be necessary to reveal principle differences in brain morphology between dog breeds.

We also not consider the influence of obesity. The fat free body weight would have been the ideal parameter to show the precise allometric change of the brain tissues.

The present study indicates that ventriculomegaly in brachycephalic dogs without morphological abnormalities of the brain parenchyma is linked to a reduction in WM volume in relation to the GM mass of the cerebrum. As the constant relationship between WM and GM is universal in all mammalian brains, the reduced WM in combination with it the larger ventricles in these dogs should not be considered a physiological condition.

The origin of the WM loss is not clear. A primary white matter disease and distension of the ventricles “ex vacuo” would theoretically be possible. Leukencephalitis, leukodystrophies, hypomyelination and spongy degeneration must be considered in this context. However, such diseases would cause distinct MRI-findings beyond white matter atrophy [9]. Moreover, primary white matter diseases would cause severe neurological signs, such as tremor, ataxia, which were not diagnosed in our patients.

Internal hydrocephalus can lead to progressive destruction of WM in dogs [45, 46] as a consequence of high intraventricular pressure. It is reasonable to expect that the degree of intraventricular pressure determines the speed and severity of white matter injury and thereby functional brain deficits. Moderately increased pressure or intermittently high intraventricular CSF pressure might play an important role in the pathogenesis of WM loss in dogs with ventriculomegaly as proposed for normal pressure hydrocephalus (NPH) in humans [47]. It has been suggested that in NPH intermittent CSF pressure waves can produce temporary phases of ischaemia in the periventricular white matter. The chronic cumulative effects of these ischaemic events can produce WM atrophy that does not arise until late adulthood [47, 48]. Brain perfusion studies have revealed reduced regional cerebral blood flow in the periventricular region in human patients with NPH, which has been attributed to disturbances in CSF flow [49].

Ventriculomegaly has also been attributed to disturbances in CSF dynamics in dogs by some authors [15, 50]. Impaired CSF flow can be caused by obstruction of the foramen magnum, as found in dogs with morphological changes in the cranio-cervical junction referred to as Chiari-like malformation [50, 51]. It has been proposed that impaired CSF flow can result in syringomyelia as well as ventricular dilatation [52, 53]. However, this has only been proposed for the Cavalier King Charles spaniel and the Brussels Griffon and the prevalence of Chiari-like malformation in other breeds with ventriculomegaly needs closer evaluation.

Human patients with enlarged ventricles in association with NPH show characteristic signs of neural function deficits. The cardinal symptoms of NPH are gait impairment, dementia and urinary incontinence (“Hakim triad”) [54]. However, it has also been proposed that the full symptom triad represents an advanced stage of the disease and that NPH can be diagnosed in the presence of only two or even just one of the symptoms [54]. In contrast, the association of solitary ventriculomegaly with neurological deficits in dogs has been rejected [9–11, 13]. The lack of clinical signs as a consequence of ventricular enlargement in dogs as opposed to humans may initially be striking. However, control of locomotion is far more complex in humans and the motor cortex and its connections play a substantial role even during undemanding steady-state walking [55]. The cerebral control of locomotion plays a minor role in dogs and diseases affecting the forebrain may have only minimal influence on locomotion [56]. Urinary incontinence was not reported in the dogs of this study either. Although the control of micturition involves cortical centres in carnivores, pontine micturition centres maintain function without cerebral control [56]. Signs of dementia are difficult to diagnose in dogs. In human medicine, a diagnosis of dementia will be considered based on the occurrence of distinct clinical signs. The most important criteria are memory impairment, disturbed language function, inability to carry out purposeful movement, and failure to recognize people [54]. Such brain functions cannot be assessed in dogs, at least not in a clinical setting, and the real status of canine cognitive abilities cannot be determined with absolute certainty. On the other hand, it has been shown that even severe ventricular dilation is compatible with normal physical and intellectual development in humans and that the ventricular volumes of hydrocephalic patients did not correlate well with their intelligence quotient [57, 58].

The influence of ventriculomegaly on brain function in dogs is unclear. Detailed behavioural studies of the impact of WM loss on the full functional integration of the nervous system are necessary to clarify whether ventriculomegaly might be an indication for CSF shunting procedures in dogs. If clinical or experimental data for cognitive impairment or intermittently high CSF pressure waves could be found, naturally occurring ventriculomegaly in dogs might be an interesting animal model for human NPH.

## Conclusion

Brachycephalic dogs with ventriculomegaly can have reduced cerebral white matter. As a predetermined relationship exists between WM and GM mass in the brain of mammals this aberration cannot be interpreted as a physiological condition.

## Supporting Information

S1 Table Calculated volumes of grey matter, white matter, lateral ventricles and the WM/GM ratio for each dog included into the study. (XLSX)
